# Synthesis and Characterization of CuO NPs Integrated Polyamidoxime–Sodium Alginate Hydrogel Nanocomposites for Antimicrobial Applications

**DOI:** 10.3390/polym18212712

**Published:** 2026-11-01

**Authors:** Juan José Andrade Sepúlveda, Radha Devi Pyarasani, Verónica Carrasco Sánchez, Samira Hozhabr Araghi, Jaime Tapia, Kishor Kumar Sadasivuni, Saravanan Chandrasekaran, Pandian Lakshmanan, John Amalraj

**Affiliations:** 1 Laboratory of Materials Science, Instituto de Química de Recursos Naturales, Universidad de Talca, 747, Talca 3460000, Chile; juan.andrade@alu.ucm.cl (J.J.A.S.); samira.hozhabr@utalca.cl (S.H.A.); 2 Centro de Investigación de Estudios Avanzados del Maule (CIEAM), Vicerrectoría de Investigación y Postgrado, Universidad Católica del Maule, Talca 3466000, Chile; 3 Laboratory of Nanomaterials, Centro de Investigación de Estudios Avanzados del Maule (CIEAM), Universidad Católica del Maule, Talca 3466000, Chile; 4 Department of Microbiology, Faculty of Health Sciences, University of Talca, Talca 3460000, Chile; vecarrasco@utalca.cl; 5 Center for Advancing Agri-Food System Transformation (CA2FST), Talca 3460000, Chile; 6 Instituto de Química de Recursos Naturales, Universidad de Talca, 747, Talca 3460000, Chile; jtapia@utalca.cl; 7 Center for Advanced Materials, Qatar University, Doha P.O. Box 2713, Qatar; kishorkumars@qu.edu.qa; 8 Department of Chemistry, School of Engineering, Presidency University, Yelahanka, Rajanukunte, Bangalore 560119, Karnataka, India; saravanan@presidencyuniversity.in; 9 Department of Chemistry, Tagore Engineering College, Rathinamangalam, Chennai 600127, Tamil Nadu, India; plakshmanan@tagore-engg.ac.in

**Keywords:** polyamidoxime, copper oxide nanoparticles, hydrogel nanocomposites, antimicrobial properties

## Abstract

Hydrogel nanocomposites with antimicrobial properties are increasingly gaining attention to combat bacterial antimicrobial resistance (AMR). In this research, we presented the development of polyamidoxime–sodium alginate (PAO-SA)-based hydrogel beads that were developed as a platform for the incorporation of copper oxide nanoparticles (CuO NPs). Two different strategies were employed to incorporate the CuO NPs: encapsulation, PAO-SA/CuO (enc) in which a relatively higher concentration of CuO NPs was localized in the core than in the shell of the hydrogel beads, and surface incorporation PAO-SA/CuO (sup) in which the CuO NPs were predominantly localized in the shell. The prepared CuO NPs and nanocomposite hydrogels were characterized by FTIR, AAS, XRD, TGA, FESEM-EDS, and HR-TEM methods. The antimicrobial activity of the CuO NP-containing PAO–SA hydrogel nanocomposites was evaluated against *Staphylococcus aureus* and *Escherichia coli*. The synthesized CuO NPs exhibited significant antimicrobial properties, with MIC and MBC values of 125 and 250 mg/L for *S. aureus* and *E. Coli*, respectively. Furthermore, bacterial growth kinetics demonstrate that the surface-supported PAO-SA/CuO (sup) nanocomposite exhibits superior antibacterial efficacy compared to its encapsulated counterpart. The in silico cytotoxicity was calculated; the IC_50_ values were approximately 24.4 mg L^−1^ for A549, 22.3 mg L^−1^ for BEAS-2B, 28.3 mg L^−1^ for MRC-5, and 40.5 mg L^−1^ for HaCaT. Overall, these results indicate that the predicted IC_50_ values are within a comparable range to the experimentally reported values for CuO nanoparticles. These results indicate that surface incorporation of CuO NPs into PAO-SA/CuO (sup) nanocomposites can be used effectively as an antimicrobial material to address bacterial AMR.

## 1. Introduction

Antimicrobial resistance (AMR) is considered one of the most serious threats to global public health. According to the 2024 Lancet report, around 7.7 million deaths are associated with bacterial infections, of which 4.95 million are associated exclusively with antimicrobial resistance. To raise awareness, the WHO estimated that AMR may cause 350 million deaths by 2050 if the problem is not addressed properly. AMR is growing in prevalence on a global scale due to intrinsic mutations, survival pressure against the excessive use of antibiotics, and horizontal gene transfer of antibiotic resistance genes. Unfortunately, in the last 40 years, there have been no new or potent versions of clinical antibiotics developed, and even pharmaceutical companies have reduced their investment in developing new drugs [1].

The rapid and versatile development of nanotechnology has provided promising alternatives for combating bacterial infections. Nanomaterials have various mechanisms for destroying bacteria; among them, the most important is membrane disruption. This may occur through physical destruction of the bacterial membrane or through chemical destruction; the latter is mostly achieved by oxidation and dephosphorylation. Another mechanism is based on the redox properties of nanomaterials, which can interfere with key cellular processes, including the biological electron transport chain, ATP synthesis, and energy metabolism, thereby controlling bacterial growth [2,3]. Catalytic killing has also gained great attention due to its persistence in bacterial control. Metal oxide NPs such as CuO, ZnO, and Fe_3_O_4_ have been widely investigated for this activity [4,5,6]. They can produce reactive oxygen species (ROS), such as hydroxyl radicals and superoxide radicals, to disturb metabolism and bacterial growth. Though metal or metal oxide NPs have excellent antimicrobial properties, there are serious issues like leaching, environmental persistence, and potential cytotoxicity [7,8]. So, we need to have a special medium to distribute these NPs to achieve excellent antimicrobial properties and minimize their potential adverse effects.

Hydrogels are one of the most popular and widely studied systems for antimicrobial and wound healing applications [9]. In general, hydrogels have better affinity for metal ions and nanoparticles due to their three-dimensional network, which can control their mobility and reduce metal ion leaching. In addition, they are soft, have a tendency to absorb water and other biological fluids, and provide a comforting feeling in the wounded area. Many biopolymers have been used to prepare hydrogels; among them, sodium alginate (SA), a well-known polysaccharide obtained primarily from seaweed, has attracted considerable attention. It can form hydrogels through ionic crosslinking with divalent ions, especially with the calcium ion (Ca^2+^), and has been used in several biomedical applications [10,11,12]. Recently, Bonini et al. demonstrated the preparation of Cu NPs-loaded SA nano fibers, and Johari et al. have developed chitosan-SA hydrogels-based Cu nanocomposites for wound dressing applications [13,14]. However, the relatively poor mechanical properties restrict their potential utility in many domains. To overcome this problem, we propose to blend SA with another synthetic polymer, such as polyamidoxime (PAO), which can be derived from well-known polyacrylonitrile (PAN). PAO has better mechanical properties and contains amidoxime functional groups, which can act as a bidentate ligand, so that it can have a stronger interaction with metal ions and potentially enhance the retention of metal species within the hydrogel matrix. This interaction can minimize metal leaching and improve the stability of the resulting hydrogel materials. PAO-based hydrogels are also well-known for their heavy metal scavenging in water treatment applications. They can effectively remove uranium ions from seawater [15,16].

It is expected that combining CuO NPs with PAO-based hydrogel materials not only improves their stability but can also make them even more effective by combining the intrinsic antimicrobial activity of CuO NPs with the structural and water-retention characteristics of hydrogels. For example, several studies have tested hydrogels with carboxymethylcellulose or chitosan mixed with CuO NPs and have shown that they can eliminate bacteria without causing significant damage to human cells [17,18,19,20]. Similarly, hydrogels with gelatin and CuO NPs have also been used for infrared light treatments, where localized photothermal heating can help to eliminate bacteria and accelerate wound healing [21]. In addition, fibers made with PAN and CuO NPs have proven to be very effective against resistant bacteria such as methicillin-resistant *Staphylococcus aureus* (MRSA) and vancomycin-intermediate *S. aureus* (VISA), even outperforming some antibiotics in laboratory tests [22,23]. These findings highlight the potential of CuO NP-loaded polymeric systems as effective platforms for developing advanced antimicrobial materials.

**Figure 1:**
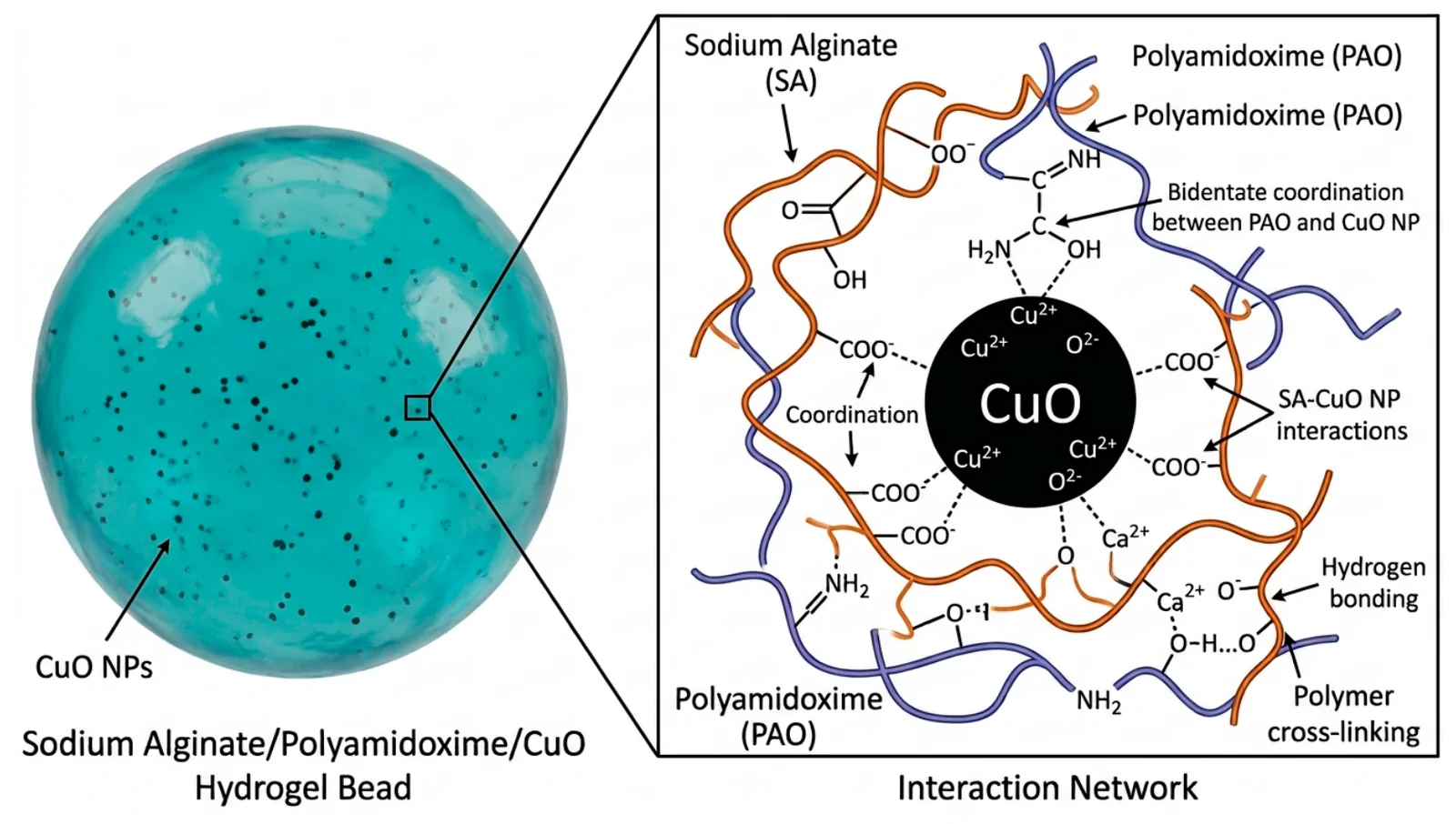
Structure of PAO-SA/CuO hydrogel bead.

In this work, we demonstrate the preparation of PAO-SA-based hydrogel beads loaded with CuO NPs (Figure 1). We prepared two types of beads: (i) hydrogel beads in which CuO NPs were encapsulated within the polymeric matrix and (ii) hydrogel beads in which CuO NPs were anchored predominantly onto the outer surface. The resulting materials were systematically characterized, and their antimicrobial properties and predicted cytotoxicity were evaluated to investigate the influence of nanoparticle localization within the hydrogel structure on their antibacterial performance.

## 2. Materials and Methods

SA, PAN, hydroxylamine hydrochloride (NH_2_OH·HCl), sodium hydroxide (NaOH), sodium carbonate (Na_2_CO_3_), copper sulfate pentahydrate (CuSO_4_·5H_2_O), dimethylformamide (DMF), calcium chloride (CaCl_2_), sodium dihydrogen phosphate monohydrate (NaH_2_PO_4_·H_2_O) and disodium hydrogen phosphate dihydrate (Na_2_HPO_4_.2H_2_O), hydrogen peroxide, and nitric acid were purchased from Sigma-Aldrich, Chile, and used as received without further purification. Milli-Q water was obtained from the Heal Force Smart ultra-pure water system.

### 2.1. Functionalization of PAN

We functionalized PAN by following a previously reported procedure with some modifications [24]. In a typical reaction, 3.1 g of NH_2_OH·HCl was dissolved in 28 mL of DMF in a two-neck round-bottomed flask. This solution was kept under constant stirring at 45 °C for a period of 1 h. Then, 1.8 g of Na_2_CO_3_ and 0.45 g of NaOH were added to the above solution and kept under constant stirring for another 2 h. Subsequently, 1 g of PAN was added to the above-mentioned solution, and the temperature was raised to 65 °C and incubated for 24 h. After 24 h, a second portion of 0.9 g of Na_2_CO_3_ and 0.22 g of NaOH was added and maintained under the same conditions for another 12 h. After completion of the reaction, the reaction mixture was brought to ambient conditions, and distilled water was added to precipitate the product. Then, the obtained precipitate was filtered, washed with an ample amount of water to remove residual reagents and solvent, and finally dried in a vacuum oven at 60 °C until reaching a constant weight.

### 2.2. Synthesis of CuO NPs

The CuO NPs are synthesized by our earlier reported procedures with modifications [18,19]. In a beaker, 1 g of CuSO_4_·5H_2_O was dissolved in 20 mL of distilled water, and the solution was heated to 85 °C and kept under constant stirring for a period of 1 h. Then, the pH was adjusted to basic conditions by adding 10 mL of 0.8 M NaOH, and the mixture was further reacted at 85 °C for 2 h under constant stirring. After 2 h, the reaction mixture was transferred to a hydrothermal reactor, which was placed in an oven at 180 °C for 24 h. The resulting CuO NPs were obtained by vacuum filtration and dried in a vacuum oven at 60 °C for 3 h. Finally, the CuO NPs were annealed in a tubular furnace at 400 °C for 4 h.

### 2.3. Hydrogel Bead Preparation

In a typical procedure, 0.6 g of SA was dissolved in 10 mL of Milli-Q water, and 0.6 g of PAO was dissolved in 10 mL of 0.5 M NaOH. Then, both solutions were mixed and kept under constant stirring for a period of 2 h. After obtaining a homogeneous mixture, it was transferred into a 1 mL micropipette fitted with a disposable tip with an inner diameter of approximately 0.8 mm. The mixture was then dispensed dropwise into a 3 wt.% CaCl_2_ solution at a rate of 46 drops per minute to form hydrogel beads. The resulting beads were collected by simple filtration and subsequently lyophilized for further characterization. The lyophilized beads were designated as pristine PAO-SA hydrogel.

CuO NPs were incorporated into the hydrogel beads by two different methods to obtain a distinct NPs distribution. For surface immobilization, 100 mg of CuO NPs were dispersed in 100 mL of 3 wt.% CaCl_2_ crosslinking solution, allowing the nanoparticles to become deposited and anchored primarily on the outer surface of the hydrogel beads. This formulation was designated as PAO–SA/CuO NPs (sup). In the second approach, 100 mg of CuO NPs were directly added to the SA solution (0.6 g in 10 mL of Milli-Q water) before gel formation, resulting in their encapsulation within the hydrogel matrix as a core. This formulation was designated as PAO–SA/CuO NPs (enc).

### 2.4. Characterization of Materials

FTIR was recorded in a JASCO FTIR-4X spectrometer (JASCO Corporation, Tokyo, Japan). The crystallite and phase composition were investigated by powder X-ray diffraction using an XRD-6100, LabX (SHIMADZU Ltd., Kyoto, Japan). Thermogravimetric analyses were conducted using a TGA 4000 (PerkinElmer, Shelton, CT, USA). The analysis was performed using around 5 mg of the samples, which were heated from ambient temperature to 800 °C at a constant heating rate of 10 °C/min under an inert nitrogen atmosphere. The morphology and surface characteristics of the synthesized materials were examined using a ZEISS Gemini SEM 360 field-emission scanning electron microscope (ZEISS, Carl Zeiss, Germany). The microstructure and surface morphology of the prepared CuO NPs were analyzed using a high-resolution transmission electron microscope (TEM) JEOL 1200 EXII, (JEOL Ltd., Tokyo, Japan).

Quantification of CuO NPs: To determine the Cu content in the polymer samples, 10 milligrams of dry sample were weighed and subjected to acid digestion in a 100 mL beaker, using a mixture of 10 mL of HNO_3_ and 5 mL of hydrogen peroxide. The solution was then heated on a hot plate at 90 °C inside a fume hood until it reached a near-dry state, following the method of Tapia et al. [25]. Subsequently, the samples were filtered and diluted to a final volume of 50 mL with double-distilled water. Copper quantification was performed via flame atomic absorption spectroscopy (air/acetylene flame) using a spectrophotometer Solaar model 969, Unicamp (Cambridge, UK). The calibration curve was prepared using a certified copper standard (Fisher Scientific, Waltham, MA, USA) with five concentration points (R^2^ > 0.999). Determinations of Cu in the polymer samples were performed, taking into account the corresponding reagent blanks. The detection limit for Cu is 4.5 μg L^−1^. All experiments were performed in triplicate to minimize experimental variability and ensure the reproducibility of the results.

### 2.5. Swelling and CuO Liberation Study

The water uptake study for the hydrogel nanocomposites was performed using the gravimetric technique. The hydrogel beads were dried at 50 °C in a vacuum oven and then stored in a vacuum desiccator to bring the hydrogels to ambient conditions. Initially, the hydrogel beads were weighed in their dry state (W_d_), and they were immediately transferred to a beaker containing Milli-Q water. A similar study was performed in phosphate-buffered solution with pH 7.4 to check the stability of the hydrogel composite in physiological condition in which the hydrogel composites with known mass were added to the freshly prepared phosphate-buffered solution and they are incubated at 37 °C in a Zhcheng shaker (model ZWY-240) with a 120 revolutions per minute (rpm).

Then, at regular intervals of time, samples were taken out, surface moisture was removed with soft tissue paper, and the weight of the swollen hydrogel beads (W_ts_) was noted. The water uptake property was calculated by the following equation:

(1) $$\mathrm{Water}\;\mathrm{uptake}\;\mathrm{capacity}\;(\%)\;=\;\frac{W_{\mathrm{ts}}-W_{d}}{W_{d}}\times 100$$

where W_ts_ and W_d_ denote the weights of the swollen hydrogels and dry hydrogels, respectively; the result is the average value of five individual sample readings.

Similarly, a CuO liberation study was performed; at predetermined time intervals, aliquots of the release medium were collected and analyzed by AAS to determine the released Cu concentration. Each withdrawn aliquot was replaced with an equal volume of fresh phosphate buffer to maintain a constant total volume. The cumulative amount of released Cu was calculated by accounting for both the dilution caused by sample withdrawal and the volume of the release medium.

### 2.6. Study of Antibacterial Activity of CuO NPs and MIC Determination

The antibacterial activity of synthesized CuO NPs was tested against the Gram-positive strain *Staphylococcus aureus* (ATCC 25923) and the Gram-negative strain *Escherichia coli* (ATCC 25922), obtained from the culture collection of the Department of Microbiology, Faculty of Health Science, Universidad de Talca, Chile.

For the first screening, antimicrobial activity was determined using a diffusion assay on Müeller–Hinton agar dishes. For this, the microorganisms were grown in Müeller–Hinton broth (MHB) overnight at 37 °C. Then, the organisms in the broth were adjusted to 0.5 McFarland turbidity (≈1.5 × 10^8^ CFU mL^−1^) and were immediately swabbed uniformly across the surface of the agar plates to create a lawn. A drop of a saturated solution of CuO NPs was placed on filter paper, and then it was placed on the grass seed, and the tests were carried out in triplicate. The plates were left incubating at 37 °C for 24 h, and then the areas that exhibited microbial growth inhibition were visually examined and measured.

To determine the minimum inhibitory concentration (MIC), a broth microdilution method was performed in 96-well microplates using MHB. The CuO NPs dispersion was diluted in geometric progression to achieve final concentrations ranging from 0 to 500 µg mL^−1^; each well was inoculated with Mueller–Hinton Broth and a bacterial strain at a concentration of 1.5 × 10^8^ CFU mL^−1^. Bacteria were incubated at 37 °C for 24 h. After incubation, the MICs were determined as the lowest nanoparticle concentrations that inhibited visible growth of microorganisms. Finally, 100 μL of each sample was added to the count agar plate to perform a colony count and determine the minimum bacterial concentration (MBC).

### 2.7. Bacterial Growth Rate Determination in the Presence of PAO-SA-CuO Beads

A growth rate curve was prepared for both *E. coli* and *S. aureus*, representing Gram-negative and Gram-positive bacteria. Briefly, tubes containing 1 mL of Müeller–Hinton broth were inoculated with the respective bacterial culture (≈1.5 × 10^8^ CFU mL^−1^). Following this, four hydrogel beads each comprising PAO-SA, PAO-SA-CuO (sup), PAO-SA-CuO (enc), and a positive control bacterium without CuO NPs were added to the separate culture tubes before incubation in a rotary incubator at 37 °C at 120 rpm. Similarly, as a negative control, only culture medium was examined. The optical density at 600 nm (OD_600_) was measured immediately after inoculation and subsequently at 12 h intervals throughout the incubation period.

### 2.8. In Silico Cytotoxicity Prediction

A web-based platform called NanoToxRadar [26,27] (https://www.kitox.re.kr/nanotoxradar, accessed on 1 September 2026) was used to predict the cytotoxicity of CuO nanoparticles. The nanoparticle composition (CuO as the core material) and an average particle diameter of 30 nm were used as input parameters, which were obtained by SEM analysis, while the other fields, including shell, doping, and coating, were left blank. This platform predicts cytotoxicity across 110 cell lines and reports the results as pIC_50_ values, defined as the negative log base 10 of IC_50_, where IC_50_ is expressed in mol L^−1^. For comparison with experimentally reported cytotoxicity data, selected predicted pIC_50_ values were converted back to IC_50_ and expressed in mg L^−1^ using the molar mass of CuO (79.55 g mol^−1^). For the NanoToxRadar analysis, CuO was used as the representative nanoparticle composition based on the predominant phase identified by the complementary characterization results.

## 3. Results and Discussion

**Figure 2:**
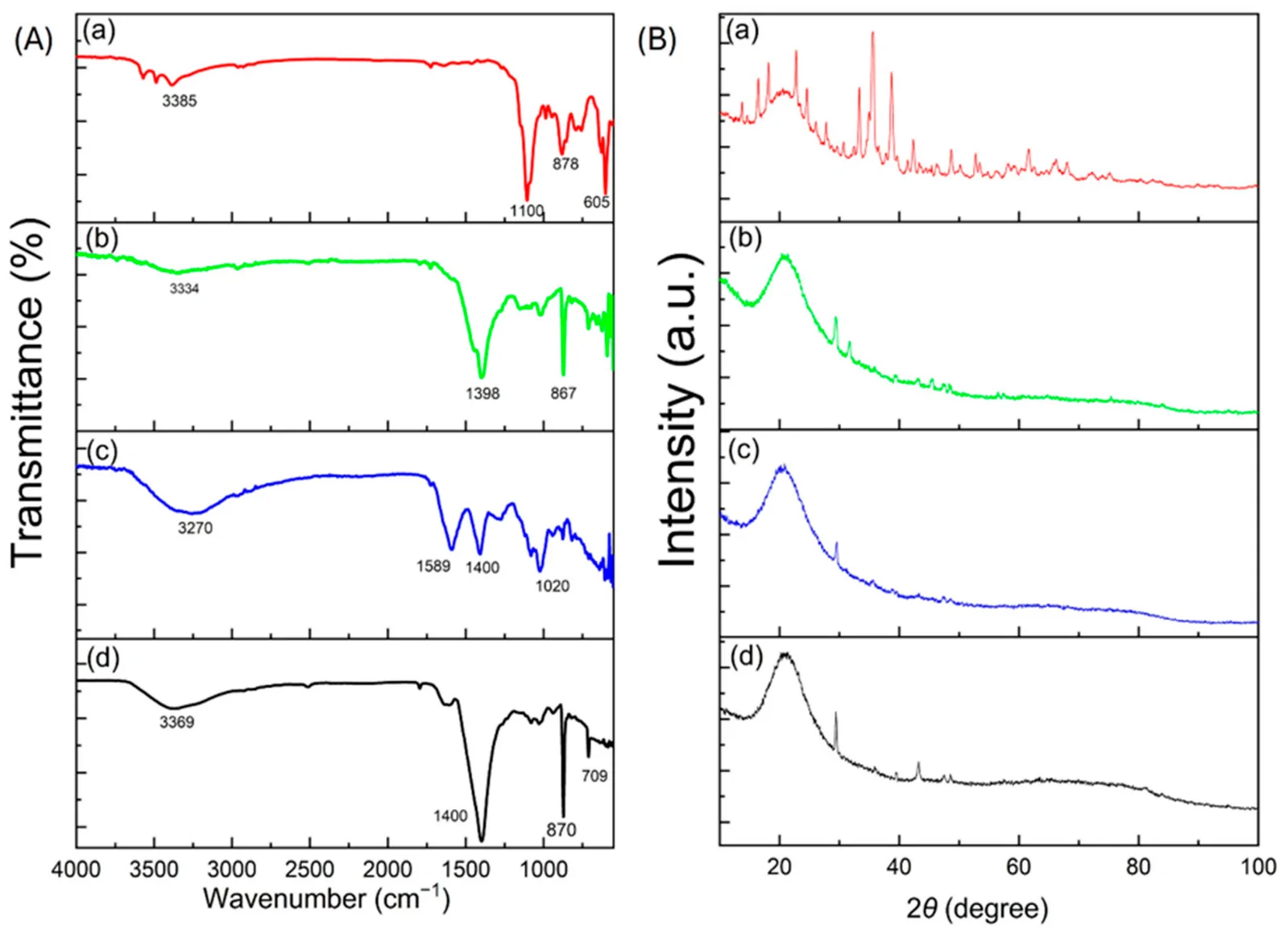
(**A**) (**a**) FTIR spectrum of (**a**) Cu NPs, (**b**) PAO-SA, (**c**) PAO-SA-enc, (**d**) PAO-SA sup. (**B**) XRD pattern of (**a**) Cu NPs, (**b**) PAO-SA, (**c**) PAO-SA- enc, (**d**) PAO-SA sup.

The FTIR spectra of the CuO NPs, pristine PAO-SA hydrogel, and CuO NPs-loaded PAO-SA hydrogel formulations are shown in Figure 2A. The FT-IR spectrum of the CuO NPs (Figure 2A-a) exhibited a strong band at 605 cm^−1^ which could be attributed to the formation of CuO NPs, and low-wavenumber bands in the ~500–650 cm^−1^ region are commonly associated with Cu–O stretching vibrations, supporting the formation of CuO NPs [28,29]. The prominent bands observed at 878 and 1100 cm^−1^ can be attributed to the vibrational modes of carbonate ions (CO_3_^2−^), likely formed through the interaction of atmospheric CO_2_ with the sample during the calcination process. These bands correspond to the in-plane bending and out-of-plane bending, respectively. Formation of the carbonate ion could occur during the calcination process. A broad absorption band at 3385 cm^−1^ can be attributed to O–H stretching vibrations associated with surface hydroxyl groups and/or adsorbed moisture.

The pristine PAO-SA hydrogel (Figure 2A-b) exhibited a broad O–H stretching band at approximately 3334 cm^−1^, together with characteristic bands around 1398 and 867 cm^−1^, corresponding to functional groups associated with the polymer matrix. In comparison, the FTIR spectrum of PAO-SA/CuO (enc) (Figure 2A-c), in which the CuO NPs were encapsulated within the polymer matrix, exhibited noticeable changes in the characteristic absorption bands. In particular, the O–H stretching band shifted from 3334 to 3270 cm^−1^, indicating a modification of the hydrogen-bonding environment of the polymer. In addition, distinct bands were observed at approximately 1589 and 1400 cm^−1^, which can be attributed to the asymmetric and symmetric stretching vibrations of carboxylate (–COO^−^) groups, respectively. The band around 1020 cm^−1^ may also be associated with changes in the oxygen-containing functional groups of the polymer network. These spectral changes suggest that the incorporation of CuO NPs within the polymer matrix modifies the local chemical environment of the hydroxyl and carboxylate groups, indicating possible interactions between the CuO NP surface and the functional groups of the polymer network. In contrast, the FTIR spectrum of PAO-SA/CuO (sup) (Figure 2A-d), in which the CuO NPs were predominantly associated with the hydrogel surface, showed considerably smaller changes in the characteristic polymer bands. The O–H stretching band was observed at 3369 cm^−1^, while the band at 1400 cm^−1^ remained close to that of the pristine PAO-SA hydrogel (1398 cm^−1^). Similarly, the band near 870 cm^−1^ was comparable to the band at 867 cm^−1^ in the pristine hydrogel. These relatively minor changes indicate that the bulk chemical environment of the polymer was less affected in PAO-SA/CuO (sup). Therefore, the FTIR results suggest that CuO NPs encapsulated within the polymer matrix in PAO-SA/CuO (enc) have a greater interaction with the polymer functional groups, whereas surface-associated CuO NPs in PAO-SA/CuO (sup) exhibit comparatively limited interaction with the bulk polymer matrix. Overall, the FTIR results are consistent with the proposed difference in CuO NP distribution within the two formulations. PAO-SA/CuO (enc) shows greater modification of the polymer functional groups, suggesting closer interaction between the CuO NPs and the polymer network, whereas PAO-SA/CuO (sup) retains FTIR features more similar to the pristine PAO-SA hydrogel, suggesting predominantly surface localization with weaker interaction with the bulk polymer matrix.

Crystal phases of CuO NPs were analyzed by XRD (Figure 2B-a), which confirms the formation of mixed oxidation states of copper NPs, such as 0, 1, and 2. Peaks occurring at 43.4 (111), 50.3 (200), and 74.1 (220) correspond to the planes of Cu NPs, 32.5 (110), 35.5 (−111) 38.7 (111) and 61.5 (−113) and 36.5 (111), 42.3 (200), 61.4 (220), and 73.5 (311) planes correspond to Cu_2_O NPs [29,30,31]. It was observed that the peak intensity of CuO was more dominant than the peaks of Cu NPs, which shows that Cu NPs are not so stable, and further oxidation leads to the formation of oxides. However, in hydrogel composites, as shown in Figure 2B-b–d, the peaks of hydrogel dominate as the concentration of CuO NPs is very low. The Debye–Scherrer equation was used to measure the average crystallite size of the CuO NPs.

τ = k λ/β cosθ(2)

where τ is the mean crystallite size, K is a dimensionless form factor (in this work, the value of 0.9 was used), λ is the wavelength of the incident X-rays, β is the width at the mean peak height (FWHM) (Figure S1), and θ is the diffraction angle. This analysis showed that the average crystallite size was 16.30 nm.

**Figure 3:**
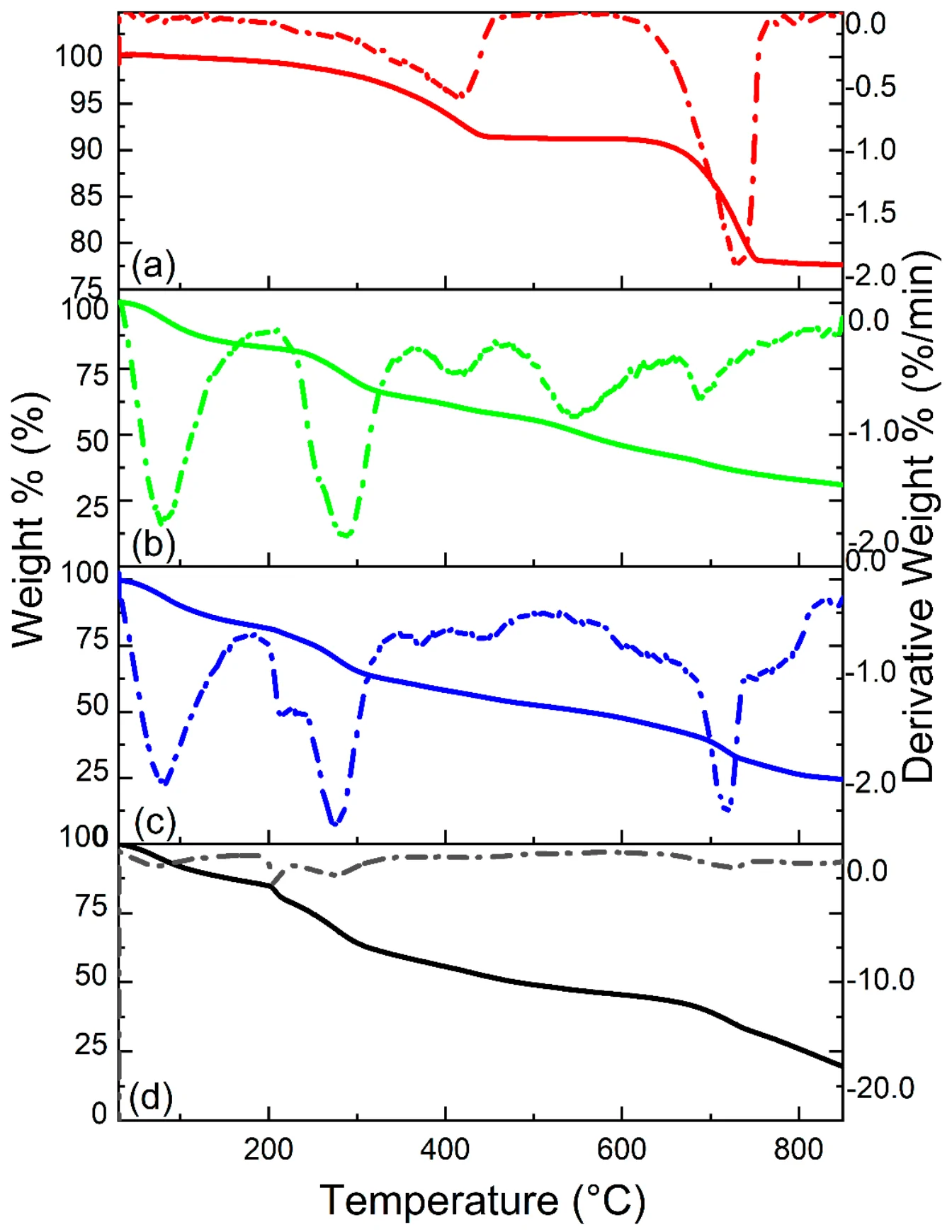
TGA and DTA spectra of (**a**) CuO NPs, (**b**) Pristine PAO-SA, (**c**) PAO-SA/CuO (enc), (**d**) PAO-SA/CuO (sup).

TGA analysis was performed to check the thermal stability, phase changes, and decomposition behavior of the synthesized components. Figure 3 displays the TGA and DTA profiles for (a) CuO NPs, (b) Pristine PAO-SA hydrogel, (c) PAO-SA/CuO (enc), and (d) PAO-SA/CuO (sup) nanocomposites from room temperature up to 850 °C. The TGA curve of the CuO NPs (Figure 3a) displayed two-step weight loss around 400 °C, 10% weight loss, and around 700 °C, another 10% weight loss; overall, it exhibits good thermal stability. A minor initial weight loss below 150 °C is attributed to the evaporation of physically adsorbed moisture. A subsequent steady weight decrease and a sharp DTA exothermic/endothermic event around 650–750 °C correspond to phase changes or structural stabilization, yielding a high residual mass (77%) characteristic of stable metallic/oxide nanostructures.

Pristine PAO-SA hydrogel (Figure 3b) displays a multi-step thermal decomposition profile characterized by initial moisture loss below 180 °C, followed by a major degradation stage between 220 °C and 320 °C due to the cleavage of glycosidic bonds and decarboxylation of the polymer backbone. A continuous mass loss follows up to 800 °C due to organic carbonization. In contrast, the PAO-SA/CuO (enc) (Figure 3c) and PAO-SA/CuO (sup) (Figure 3d) nanocomposites exhibit modified thermal profiles, featuring shifted degradation temperatures and altered residual masses due to the presence of CuO NPs. A prominent DTA peak near 700 °C corresponds to advanced pyrolysis, carbonization, and the thermal decomposition of calcium–polymer complexes. This peak is noticeably sharper and more pronounced in the encapsulated composite (Figure 3c) than in the surface-anchored composite (Figure 3d). This difference stems from spatial confinement: the densely trapped nanoparticles and crosslinked core in PAO-SA/CuO (enc) cause a concentrated heat flow event during structural collapse, whereas the surface is more accessible. We could not analyze the weight content of the CuO NPs by analyzing the residual mass, since we used CaCl_2_ as a crosslinker, which would affect this analysis.

### 3.1. Quantification of CuO NPs in Hydrogels

The CuO content of the PAO-SA/CuO hydrogel nanocomposites was quantitatively determined by atomic absorption spectroscopy (AAS). The AAS analysis revealed CuO contents of 33.7 wt% and 29.0 wt% for PAO-SA/CuO-Enc and PAO-SA/CuO-Sup, respectively. These results confirm the incorporation of copper-containing species within the hydrogel matrix.

### 3.2. Swelling Study

**Figure 4:**
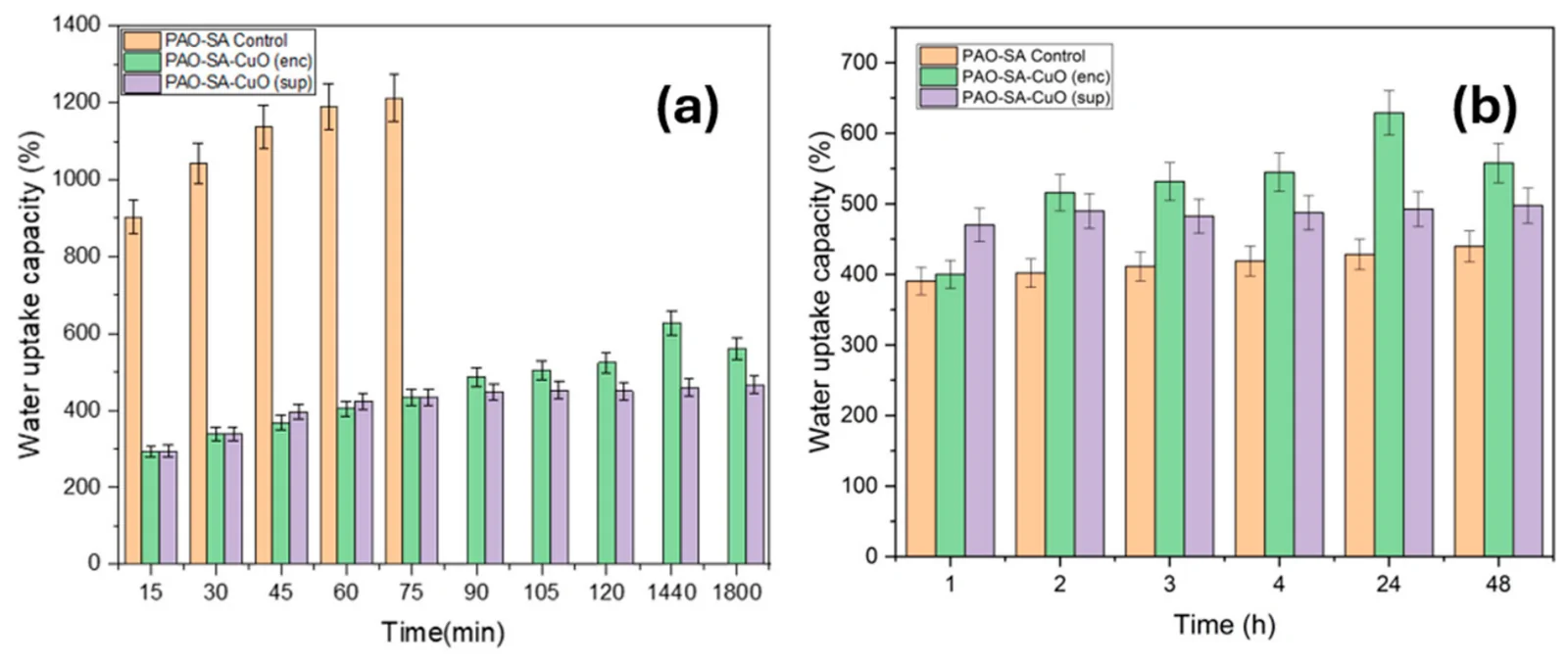
Water uptake analysis of pristine PAO–SA hydrogel (PAO–SA Control), PAO–SA-CuO (enc), and PAO–SA-CuO (sup) as a function of immersion time in water (**a**) and phosphate buffer (**b**).

**Figure 5:**
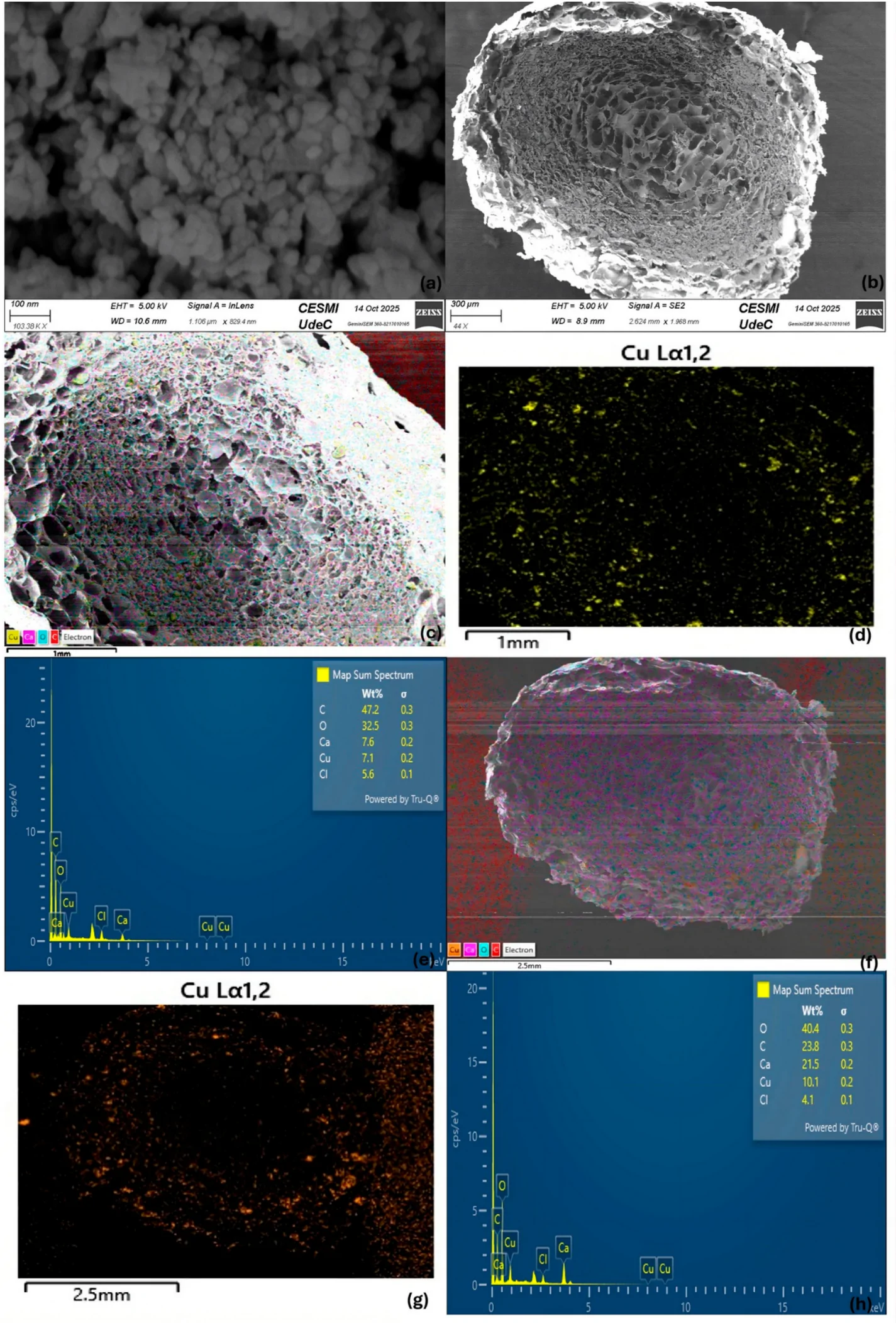
SEM analysis of (**a**) CuO NPs, (**b**) PAO-SA, (**c**) PAO-SA/CuO (enc), (**d**) Cu metal mapping of PAO-SA (enc), (**e**) elemental analysis spectrum of PAO-SA (enc), (**f**) PAO-SA/CuO (sup), (**g**) Cu metal mapping of PAO-SA (sup), (**h**) elemental analysis spectrum of PAO-SA (sup).

The swelling behavior and water uptake capacity of the hydrogel nanocomposites as a function of time are given in Figure 4a. The pristine PAO-SA hydrogel beads exhibited rapid initial swelling, reaching approximately 900% water uptake within the first 15 min and a maximum swelling capacity of approximately 1200% at the equilibrium time of 75 min. However, prolonged immersion resulted in the gradual disintegration of the pristine hydrogel beads, which can be attributed to their relatively poor mechanical stability. Therefore, in Figure 5, pristine hydrogel is not presented after 75 min.

In contrast, the incorporation of CuO nanoparticles significantly reduced the initial swelling rate while improving the structural stability of the hydrogel beads. The PAO-SA/CuO (sup) hydrogel exhibited approximately 300% water uptake after 15 min, followed by a gradual increase to approximately 466% after 30 h. Similarly, the PAO-SA/CuO (enc) hydrogel reached approximately 294% water uptake within 15 min and increased progressively to approximately 562% after 30 h. The slightly higher water uptake observed for PAO-SA/CuO (enc) compared with PAO-SA/CuO (sup) may be attributed to differences in the distribution of CuO nanoparticles within the hydrogel matrix. In particular, the presence of excess CuO nanoparticles near the surface of the PAO-SA/CuO (sup) beads may restrict water penetration and consequently decrease their overall water uptake.

Although the incorporation of CuO nanoparticles decreases the maximum swelling capacity compared with the pristine PAO-SA hydrogel, it markedly enhances the stability of the hydrogel network. This behavior suggests that CuO nanoparticles interact with the polymer chains and act as additional cross-linking points within the PAO-SA matrix, thereby restricting excessive swelling and preventing rapid disintegration. Thus, the incorporation of CuO provides a balance between water absorption and structural integrity, resulting in hydrogel nanocomposites with improved stability during prolonged immersion.

A similar study was performed in phosphate buffer with pH 7.4 (Figure 4b) to check the stability of the hydrogel composites in the physiological condition. Interestingly, PAO-SA hydrogel beads had a relatively lower water uptake around 400% even after 48 h; however, they show better stability than in pure water. It may be attributed to the presence of potassium, phosphate ions may modify electrostatic interactions, and osmatic swelling may decrease the calcium ion leaching and provide additional stability by interacting with amidoxime groups in the hydrogel. PAO-SA-CuO (enc) and PAO-SA-CuO (sup) have shown a slightly better water intake capacity around 600%, and CuO incorporation may change the pore structure, polymer packing and hydrophilic environment. After 48 h, both PAO-SA-CuO (enc) and PAO-SA-CuO (sup) hydrogel composites began to disintegrate, and complete disintegration was observed after 72 h (Figure S2).

The release kinetics of CuO from the hydrogel nanocomposites were investigated using zero-order, first-order, Higuchi, and Korsmeyer–Peppas models (Table 1). For the PAO-SA/CuO (sup) system, the Korsmeyer–Peppas model exhibited the highest coefficient of determination (R^2^ = 0.8581), followed by the Higuchi model (R^2^ = 0.7983). Similarly, for the PAO-SA/CuO (enc) system, the Korsmeyer–Peppas model showed a higher (R^2^) value (0.9149) than the Higuchi model (R^2^ = 0.8801). The release exponents (*n* = 0.176 and 0.2273) for the PAO-SA/CuO (sup) and PAO-SA/CuO (enc) systems, respectively, suggest a release profile that is predominantly governed by diffusion-related processes. It was observed that even after 48 h, only around 30% of CuO has been liberated; the relatively slow CuO liberation may be associated with strong interactions between CuO and amidoxime groups which are presented in the hydrogel network.

### 3.3. SEM and TEM Analysis

Field emission scanning electron microscopy (FE-SEM) analysis was performed to examine the surface morphology, structural organization, and elemental distribution of the synthesized CuO NPs and the polymer-alginate (PAO-SA) hydrogel nanocomposites. As shown in Figure 5a, the high-magnification FE-SEM micrograph of the synthesized CuO NPs reveals densely packed, nearly spherical nanoparticles with an average size of approximately 30 nm. The observed tendency toward nanoparticle agglomeration is characteristic of metallic nanostructures synthesized via hydrothermal approaches, driven by their high surface area-to-volume ratio and high surface free energy [32,33].

The structural morphology of the hydrogel beads is illustrated in Figure 5b,c. Figure 5b shows the PAO-SA hydrogels with a hollow structure in the middle and a compact structure at the shell, which might be attributed to the diffusion-controlled crosslinking mechanism of divalent calcium ions during gelation. Rapid ionic crosslinking predominantly occurs at the outer boundary upon initial contact with the CaCl_2_ solution, forming a dense skin layer, whereas the interior experiences restricted diffusion, resulting in a looser, highly porous, or hollow core network [34]. Similar core–shell or heterogeneous structures have been widely documented in ionic-crosslinked alginate-based composite hydrogels [35,36].

Furthermore, elemental mapping and energy-dispersive X-ray (EDX) spectroscopy (Figure 5d) confirmed the successful incorporation and distribution of CuO NPs throughout the hydrogel matrix. The CuO metal mapping demonstrates that the nanoparticles are dispersed without massive phase-segregated macroscopic clustering. Quantitative FE-SEM-EDX analyses revealed that the supported (PAO-SA/CuO sup) and encapsulated (PAO-SA/CuO enc) hydrogel composites contain 10 wt.% (Figure 5f) and 7.1 wt.% (Figure 5e) of CuO NPs, respectively.

**Figure 6:**
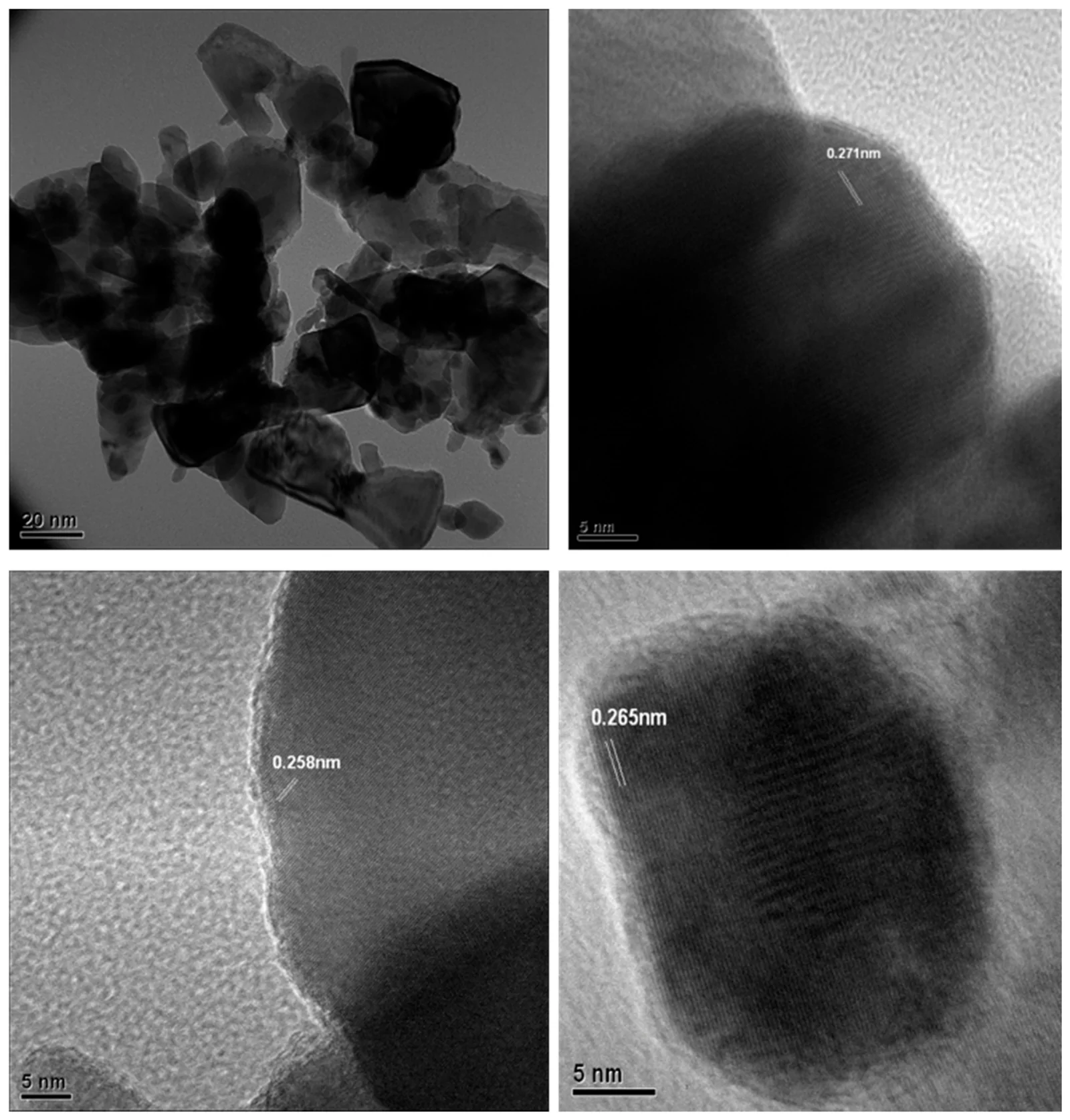
HR-TEM analysis of CuO NPs.

The HR-TEM images of CuO NPs are shown in Figure 6. The HR-TEM images confirmed the formation of monodisperse CuO nanoparticles, and the HR-TEM images show the CuO crystalline lattice fringes with the interplanar spacings of 0.258, 0.65, and 0.271, which correspond to the (020), (111), and (010) facets of monoclinic CuO NPs, respectively [37].

### 3.4. Antibacterial Activity of CuO NPs and MIC Determination

**Figure 7:**
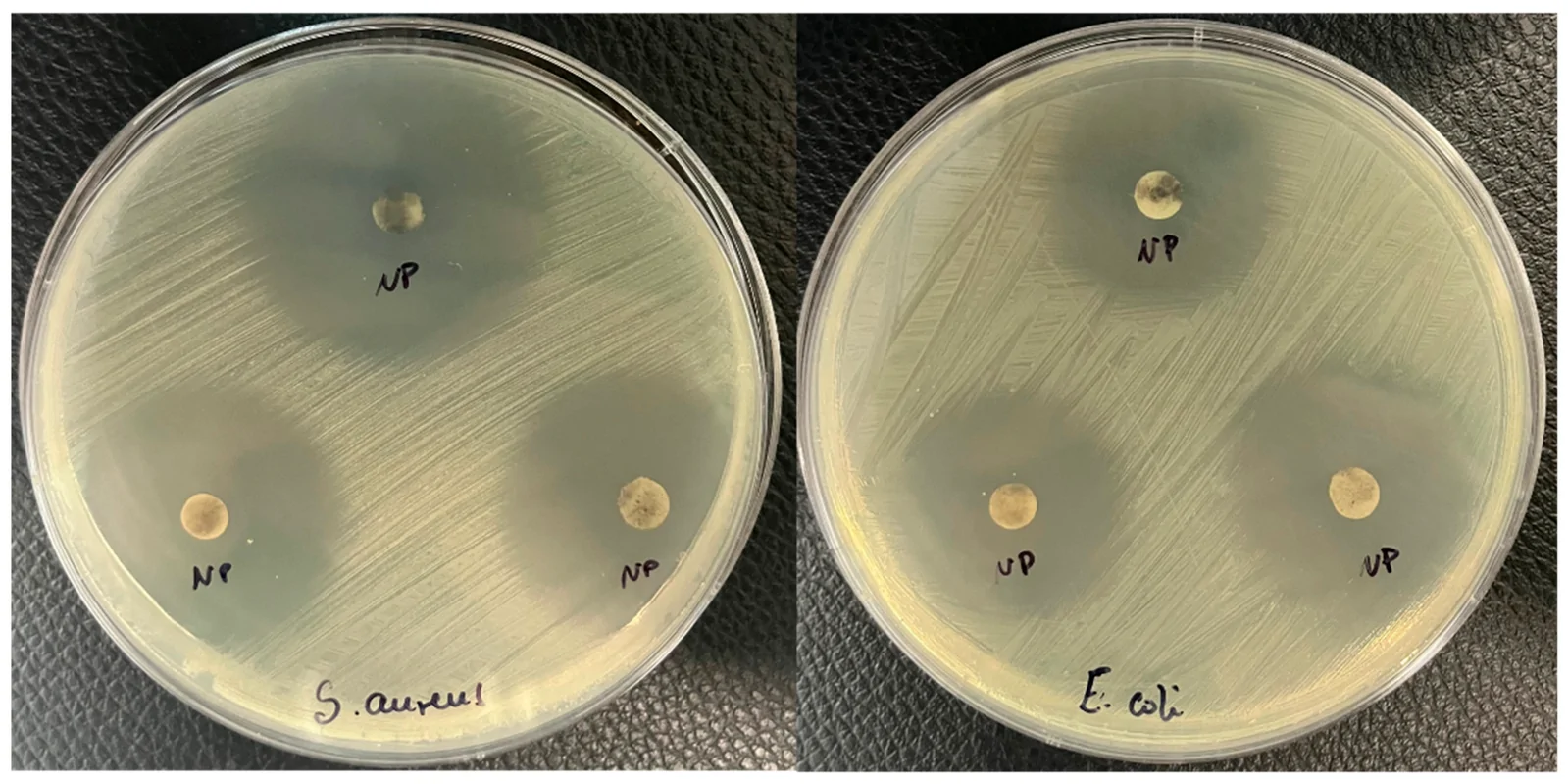
Antimicrobial screening of CuO NPs on *S. aureus* and *E. coli.*

The antibacterial activity of the CuO nanoparticles (NPs) was initially evaluated against *Staphylococcus aureus* and *Escherichia coli* using a screening assay (Figure 7). The CuO NPs exhibited clear antibacterial activity against both bacterial strains, confirming their ability to inhibit bacterial growth. The minimum inhibitory concentration (MIC) and minimum bactericidal concentration (MBC) were subsequently determined, and the results are summarized in Table 2.

The MIC of the CuO NPs was 125 mg L^−1^ for both *S. aureus* and *E. coli*, indicating that the nanoparticles were effective at inhibiting the visible growth of both Gram-positive and Gram-negative bacteria at the same concentration. The corresponding MBC was 250 mg L^−1^ for both bacterial strains. Thus, the MBC/MIC ratio was 2 for both *S. aureus* and *E. coli*. Since an MBC/MIC ratio ≤ 4 is generally considered indicative of bactericidal activity, these results suggest that the CuO NPs exert a bactericidal rather than predominantly bacteriostatic effect against both bacterial strains. Similar antibacterial behavior of CuO NPs has been reported previously by Khairy et al. [38].

The comparable MIC and MBC values obtained for the two bacterial strains suggest that the antibacterial activity of the CuO NPs was not strongly dependent on bacterial Gram classification under the experimental conditions used. The observed antibacterial effect may be associated with the interaction of Cu-containing nanoparticles with the bacterial cell envelope, together with the release of copper ions and the generation of reactive oxygen species, which can cause membrane damage and intracellular oxidative stress. These mechanisms can ultimately lead to loss of cellular integrity and bacterial death [2]. Overall, the results demonstrate that the synthesized CuO NPs possess significant bactericidal activity against both *S. aureus* and *E. coli*, supporting their potential incorporation into polymeric materials for antimicrobial applications.

### 3.5. Bacterial Growth Rate Determination in the Presence of Alginate-NPs Bead

**Figure 8:**
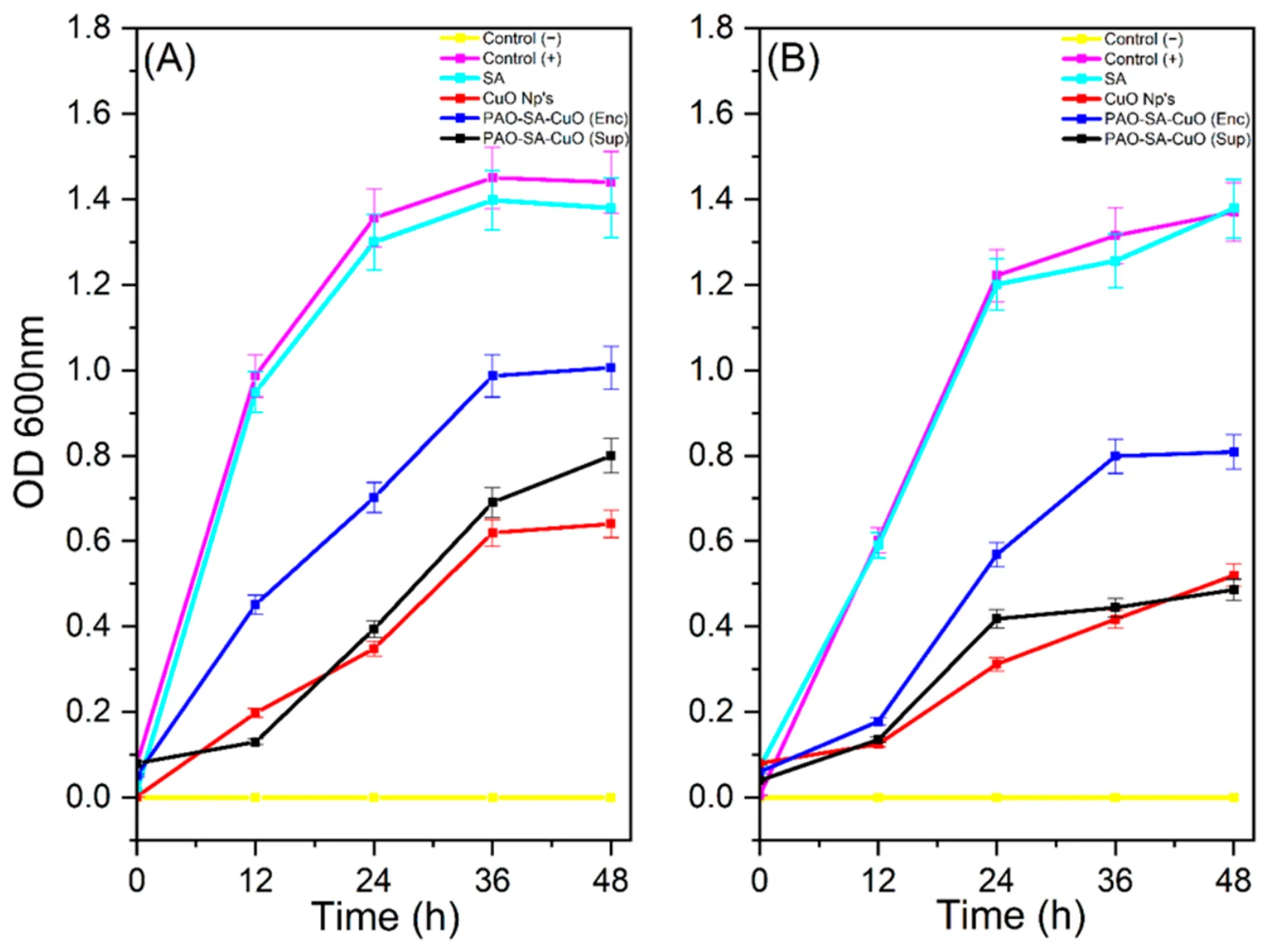
Bacterial growth curve of (**A**) *S. aureus* and (**B**) *E. coli* after treatment with the nanocomposite and its individual controls. The values are represented as mean ± SD of results from three individual experiments.

The bacterial growth inhibition curve was used to study and compare the antibacterial efficacy of the CuO NPs when incorporated on the surface and when encapsulated in the polymeric systems. The results are shown in Figure 8A,B.

Bacterial growth kinetics monitored at OD_600_ over 48 h demonstrated distinct antimicrobial profiles that were dependent on the formulation and bacterial strain (Figure 8). The negative control confirmed system sterility, whereas both the positive control and pure sodium alginate matrix (SA) exhibited uninhibited growth for *Staphylococcus aureus* (A) and *Escherichia coli* (B), reaching OD_600_ values of ∼1.4–1.45 at 48 h and confirming that SA possesses no intrinsic antibacterial activity. In contrast, free CuO nanoparticles (CuO NPs) exerted strong growth suppression, maintaining final absorbance values below 0.64 against *S. aureus* and 0.52 against *E. coli*. Among the composite materials, the surface-supported formulation PAO-SA/CuO (sup) exhibited markedly higher antibacterial efficacy than its encapsulated counterpart (PAO-SA/CuO (enc), restricting 48 h growth to OD_600_ ≈ 0.80 in *S. aureus* and matching or exceeding free CuO NPs in *E. coli* (OD_600_ ≈ 0.49). This superior performance of PAO-SA/CuO (sup) stems from direct surface contact and reduced diffusion resistance, allowing rapid active release into the culture medium. Conversely, encapsulation within the SA hydrogel network PAO-SA/CuO (enc) restricts active site accessibility, leading to a slower, diffusion-controlled release mechanism and higher late-stage optical densities (OD_600_ ≈ 1.01 for *S. aureus* and 0.81 for *E. coli*). The observed inhibition across the treated groups is driven by ROS generation and Cu^2+^ ionic release, which disrupt bacterial cell membrane integrity. Furthermore, the higher overall susceptibility observed in *E. coli* compared to *S. aureus* reflects structural differences between Gram-negative and Gram-positive cell walls in restricting nanoparticle and ion penetration.

### 3.6. In Silico Cytotoxicity Prediction of CuO NPs

**Figure 9:**
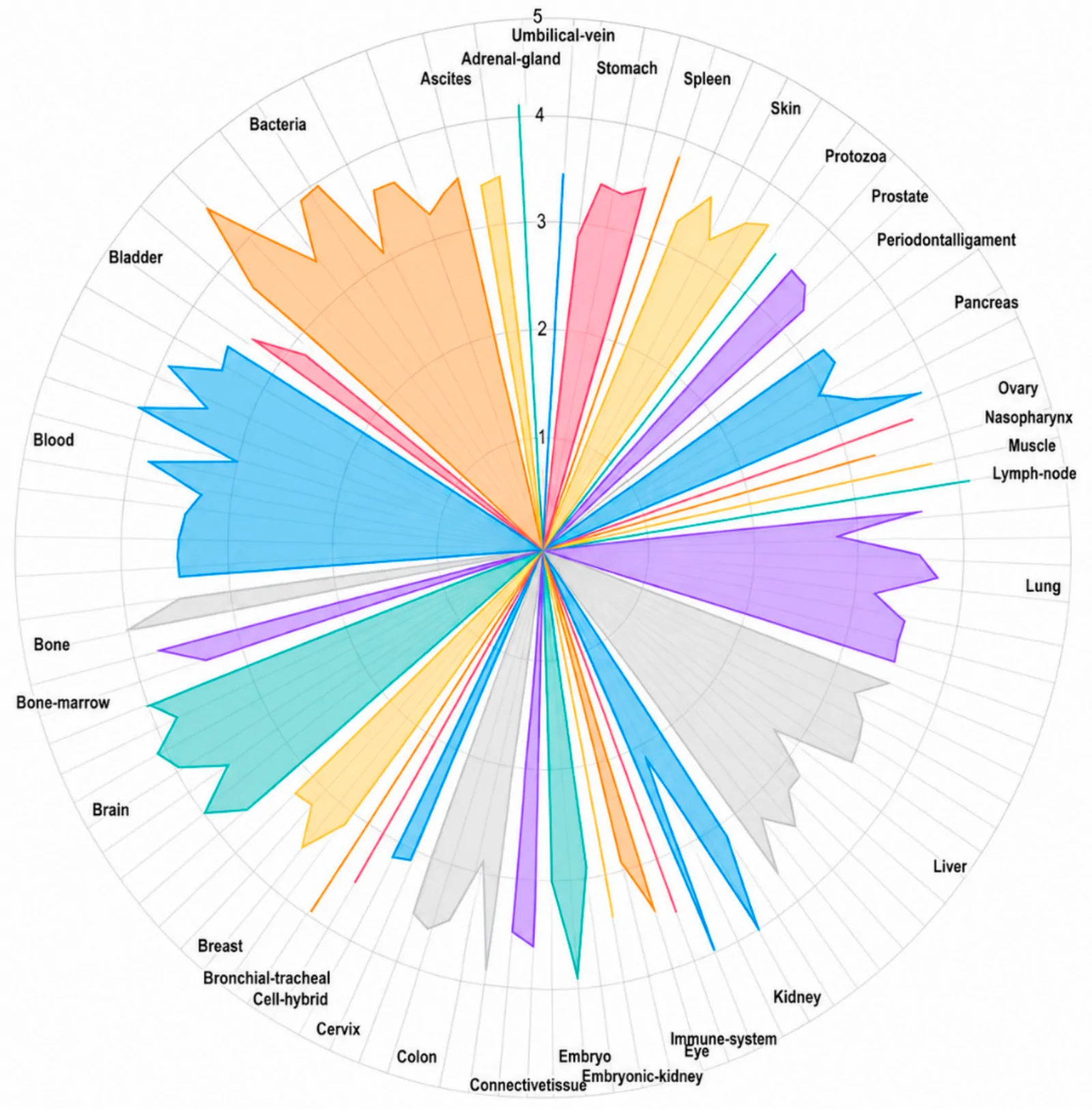
Radar plot depicting the predicted pIC_50_ distribution of CuO nanoparticles.

Since the NanoToxRadar output included predicted pIC_50_ values for a large number of cell lines (see Figure 9 and Table S1), a literature review was conducted to select a limited number of cell lines for cytotoxicity analysis. It focused on studies that experimentally evaluated the cytotoxicity of CuO nanoparticles with particle sizes similar to the nanoparticles investigated in the present study. Based on this review, A549 (human lung cancer cells) [39], BEAS-2B (human bronchial epithelial cells) [40], MRC-5 (human lung fibroblasts) [41], and HaCaT (human keratinocytes) [42] were selected for comparison with the predicted cytotoxicity results. The corresponding pIC_50_ values for these cell lines were extracted from the NanoToxRadar output (Table S1) and were 3.513, 3.553, 3.449, and 3.293 for A549, BEAS-2B, MRC-5, and HaCaT, respectively. After conversion, the IC_50_ values were approximately 24.4 mg L^−1^ for A549, 22.3 mg L^−1^ for BEAS-2B, 28.3 mg L^−1^ for MRC-5, and 40.5 mg L^−1^ for HaCaT. Overall, these results indicate that the predicted IC_50_ values were within a comparable range to the experimentally reported values for CuO nanoparticles of similar sizes, although some differences were observed among the cell lines.

## 4. Conclusions

In this work, PAO-SA hydrogel nanocomposites containing Cu-containing nanoparticles were successfully prepared using two different approaches, namely encapsulation within the hydrogel matrix [PAO-SA/CuO (enc)] and incorporation at the hydrogel surface [PAO-SA/CuO (sup)]. Structural and spectroscopic characterization confirmed the incorporation of copper-containing nanoparticles, with XRD analysis indicating the presence of Cu, Cu_2_O, and CuO phases. The nanoparticle contents were determined to be 33.7 wt.% for PAO-SA/CuO (enc) and 29.0 wt.% for PAO-SA/CuO (sup). The incorporation of the nanoparticles reduced the swelling capacity compared with pristine PAO-SA hydrogel but significantly improved the structural stability of the hydrogel nanocomposites during prolonged water immersion. Both Cu-containing hydrogel nanocomposites exhibited antimicrobial activity against *E. coli* and *S. aureus*, with MIC and MBC values of 125 and 250 mg/L, respectively. In silico cytotoxicity prediction of CuO NPs also revealed that the predicted IC_50_ values were within a comparable range to the experimentally reported values for CuO nanoparticles of similar sizes. Overall, the results demonstrate that CuO-containing PAO-SA hydrogel nanocomposites, particularly PAO-SA/CuO (sup), possess promising antimicrobial properties combined with improved hydrogel stability, making them potential candidates for antimicrobial material applications.

## Figures and Tables

**Table 1:** Liberation of CuO NPs from hydrogel nanocomposites.

Time	PAO-SA/CuO (Sup)		PAO-SA/CuO (Enc)	
	Concentration (mg/L)	Cumulative Liberation (Wt.%)	Concentration (mg/L)	Cumulative Liberation (Wt.%)
1	2.8	13.3	3.2	13.2
2	3.6	18.0	3.7	15.9
3	4.1	21.1	4.7	20.1
4	4.2	22.7	4.9	22.8
24	5.1	27.1	5.8	27.6
48	5.4	29.0	6.2	30.6

**Table 2:** Results of MIC and MBC of CuO NPs against bacteria.

Bacterial Strains	CuO NPs (mg/L)		
	MIC	MBC	MBC/MIC
*E. coli*	125	250	2
*S. aureus*	125	250	2

## Data Availability

The original contributions presented in this study are included in the article/Supplementary Material. Further inquiries can be directed to the corresponding authors.

## Supplementary Materials

The following supporting information can be downloaded at https://www.mdpi.com/article/10.3390/polym18212712/s1: Figure S1: Full width at half maximum (FWHM) calculation of CuO NPs; Figure S2: Stability of PAO-SA hydrogels (a) 1 h, (b) 48 h, (c) 72 h, PAO-Sa/CuO (enc) (d) 1 h, (e) 48 h, (f) 72 h, PAO-Sa/CuO (sup) (g) 1 h, (h) 48 h, (i) 72 h; Table S1: Predicted pIC50 values of 30 nm CuO nanoparticle across 110 cell lines obtained using NanoToxRadar.
