# Supplementary material for: Synthesis and Characterization of CuO NPs Integrated Polyamidoxime–Sodium Alginate Hydrogel Nanocomposites for Antimicrobial Applications
Source: Polymers (Basel). 2026 Nov 1;18(21):2712. doi: 10.3390/polym18212712 (PMC13765577; doi:10.3390/polym18212712)
Supplement: Supplementary file 1 [file polymers-18-02712-s001.zip › polymers-4577010-supplementary.pdf]

Supporting information

Synthesis and Characterization of CuO NPs Integrated Polyamidoxime–Sodium Alginate Hydrogel Nanocomposites for Antimicrobial Applications

Juan José Andrade Sepúlveda [a], Radha Devi Pyarasani [b,c,*], Verónica Carrasco Sánchez [d,e], Samira Hozhabr Araghi [a], Kishor Kumar Sadasivuni [f], Saravanan Chandrasekaran [g], Pandian Lakshmanan [h] and John Amalraj [a,*]

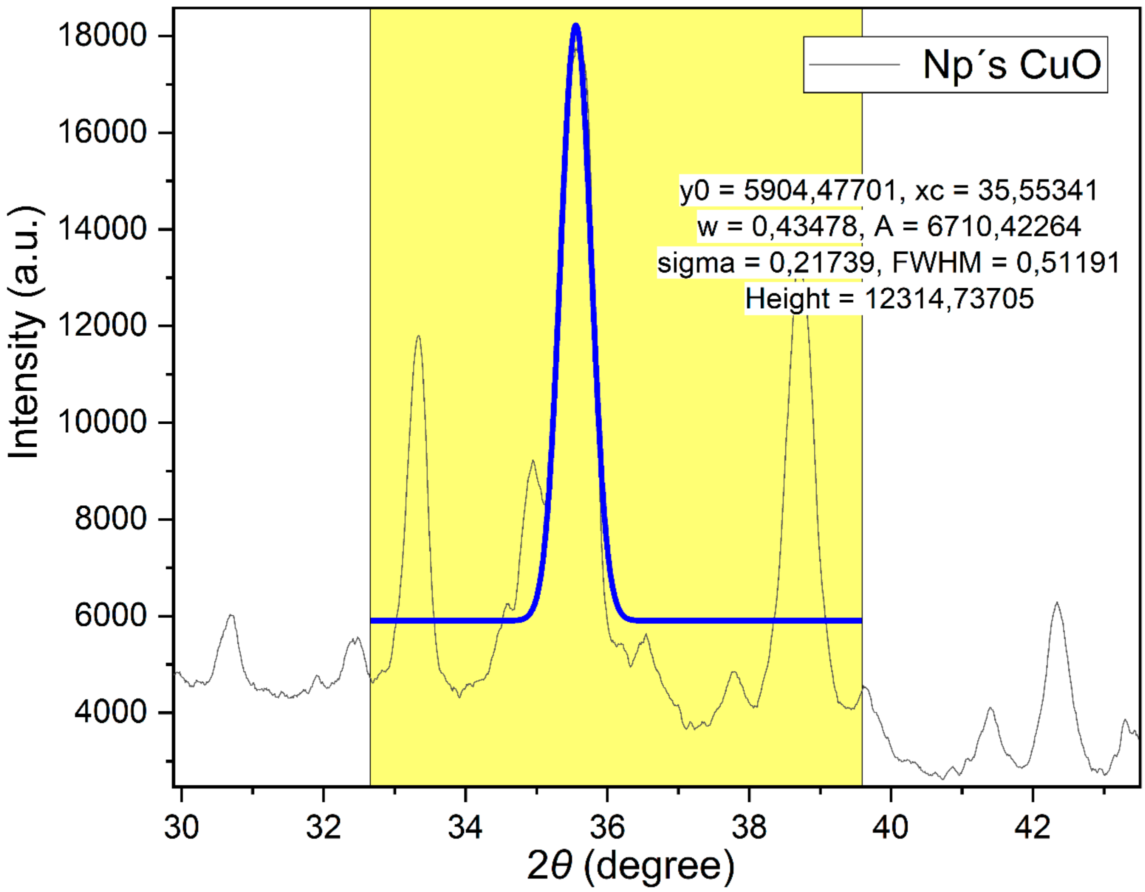

Figure S1. Full width at half maximum (FWHM) calculation of CuO NPs.

Table S1. Predicted pIC_{50} values of 30 nm CuO nanoparticles across 110 cell lines obtained using NanoToxRadar.

Cell tissue	Cell identification	Prediction
Adrenal gland	PC12	4.064
Ascites	J774	3.43
	Ehrlich Ascites Carcinoma	3.478
Bacteria	E. Coli	3.713
	Bacillus Subtilis	4.474
	S. paratyphi	3.864
	P. aeruginosa	3.424
	S. aureus	3.949
	E. hirae	3.981
	Klebsiella pneumoniae	3.136
	Listeria monocytogenes	3.678
	Proteus vulgaris	3.673
	Streptococcus pyogenes	3.269
	V. cholerae (Tolerant phenotype)	3.437
	V. cholerae (Vulnerable phenotype)	3.512
Bladder	ECV304	3.382
	MGH	2.885
Blood	K562	3.466
	RAW264-7	3.484
	Jurkat	3.449
	Trout leukocytes	3.409
	Mouse leukocytes	3.289
	Human monocytes	3.823
	THP-1	3.008
	B-LCL	4.044
	Human normal lymphocyte	3.419
	Daudi	3.929
	PBMC	3.472
	Ramos.2G6.4C10	3.523
Bone	MG-63	4.013
	Osteoblast	3.475
Bone marrow	SH-SY5Y	3.367
	hMSCs	3.751
Brain	IMR	3.661
	U251	3.997
	C17-2-Neural progenitor	3.58
	U87	3.957

	A172	4.101
	C6 glioma	3.791
	NG108-15	4.001
Breast	MCF-7	3.117
	MDA-MB-231	3.536
	SK-BR-3	3.186
	T-47D	3.231
Bronchial–tracheal	NHBE	3.934
Cell hybrid	EAhy926	3.482
Cervix	HeLa	3.074
	KB	3.133
Colon	HCT 116	3.836
	HT 29	2.889
	Caco-2	3.458
	CCD-18Co	3.598
	HCT-8 (HRT 18)	3.485
Connective tissue	L929	3.595
	HT 1080	3.461
Embryo	NIH/3T3	2.876
	BALB/3T3	3.896
	3T3	2.923
Embryonic kidney	HEK293	3.358
Eye	Bovine corneal fibroblasts	3.397
	ARPE-19	2.91
Immune system	Mussel hemocytes	3.489
Kidney	Vero	3.119
	COS	3.984
	MDCK2	2.142
	LLC-PK1	3.967
Liver	Primary human hepatocyte	3.417
	HepG2	3.206
	huh 7 cells	3.389
	Zebrafish liver cell	3.453
	HCC	3.484
	Hepatocyte	2.687
	Trout hepatocytes	3.159
	LO2	3.164
	Bel 7402	3.459
	HL-7702	3.174
	SMMC-7721	3.661
Lung	A549	3.513

	SPCA1	2.817
	BEAS-2B	3.553
	CHL	3.73
	H460	3.176
	HELF	3.469
	MRC-5	3.449
	SK-LC-6	3.478
Lymph node	Cancer stem cell/FEMX-1	4.035
Muscle	Myoblast	3.732
Nasopharynx	HK-1	3.256
Ovary	CHO-K1	3.601
Pancreas	PANC-1	3.216
	AsPC-1	3.243
	BXPC-3	2.976
	MIA PaCa 2	3.248
	Panc-28	3.817
Periodontal ligament	PDL-hTERT	3.466
Prostate	PC3	3.498
	DU-145	3.478
	HPrEC	3.29
Protozoa	T-Thermophila-BF5	3.459
Skin	HaCaT	3.293
	HT 144	3.662
	B16	3.26
	JB6	3.573
	A375	3.686
Spleen	WIL2-NS	3.835
Stomach	SGC-7901	2.888
	AGS	3.434
	KATO III	3.375
	MKN45	3.475
Umbilical vein	HUVEC	3.478

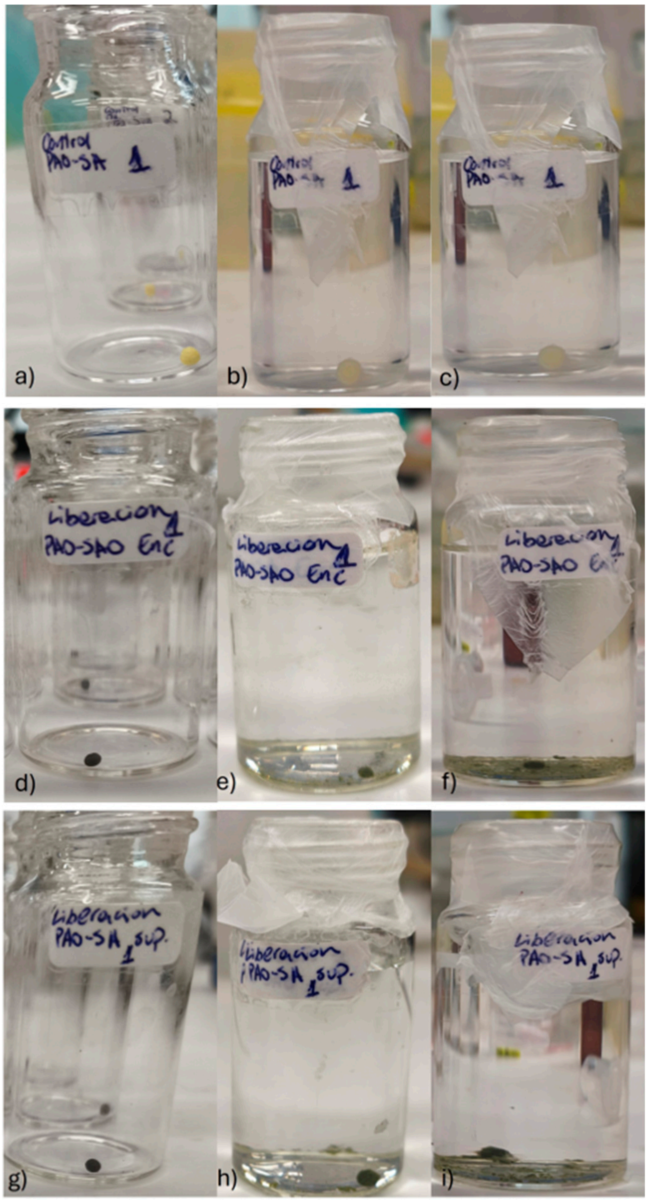

Figure S2. Stability of PAO-SA hydrogels: (a) 1 h, (b) 48 h , (c) 72 h, PAO-Sa/CuO(enc) (d) 1 h, (e) 48 h , (f) 72 h, , PAO-Sa/CuO(sup) (g) 1 h, (h) 48 h , (i) 72 h.
